# Occult hepatitis B infection in different high risk patients

**DOI:** 10.5812/hepatmon.7094

**Published:** 2012-07-30

**Authors:** Amitis Ramezani, Mohammad Banifazl, Ali Eslamifar, Masoomeh Sofian, Arezoo Aghakhani

**Affiliations:** 1 Clinical Research Department, Pasteur Institute of Iran, Tehran, IR Iran; 2 Pediatric Infectious Disease Research Center, Tehran University of Medical sciences, Tehran, IR Iran; 3 Iranian Society for Support of Patients With Infectious Diseases, Tehran, IR Iran; 4 Tuberculosis and Pediatric Infectious Research Center (TPIRC), Arak University of Medical Sciences, Arak, IR Iran

**Keywords:** Hepatitis B, Renal Dialysis, HIV

Dear Editor,

We read with great interest the article “epidemiology of occult hepatitis B infection among thalassemia, hemophilia and hemodialysis patients” by Arababadi et al. [1]. because of our common field of interest in investigation of occult hepatitis B infection (OBI) in high risk patients such as HIV infected cases and hemodialysis patients. OBI is defined as the presence of HBV-DNA in the liver tissue or serum without detectable hepatitis B surface antigen (HBsAg) [2]. About 20 % of OBI cases are negative for all HBV markers except HBV-DNA, 50 % are positive for hepatitis B core antibody (anti-HBc) and 35 % are positive for hepatitis B surface antibody (anti-HBs) [3]. We investigated OBI in HIV positive patients [10][11]. OBI is an important issue in these patients because HIV/HBV co-infected individuals are at increased risk of chronic hepatitis, cirrhosis, and hepatocellular carcinoma [4]. In the published reports, the prevalence of occult HBV infection in HIV infected patients ranged between 0-10 % using standard PCR methods [5][6][7] and 35- 89 % using more sensitive assays [8][9] . In our study, out of 106 enrolled HIV infected patients, 20.75 % had isolated anti-HBc (HBsAg negative, anti-HBs negative and anti-HBc positive). HBV-DNA was detected in 13.6 % of patients with isolated anti-HBc. We divided these patients based on their HCV status. Out Of 63 anti-HCV positive cases, 28.6 % had isolated anti-HBc and 16.7 % of the latter group had OBI. But we did not find any OBI in HIV patients without HCV infection [10][11]. We also conducted the same study in hemodialysis patients [12]. The prevalence of occult HBV infection in dialysis patients was reported with a range between 0 to 58 % in published studies [13][14][15][16]. We found that 6.2 % of 289 enrolled patients had isolated anti-HBc and OBI was detected in 50 % of these patients. Only one of our patients with occult HBV was co-infected with HCV, so a conclusion cannot be reached regarding the association of occult HBV infection and HCV in this study [12].

Overall, the rate of OBI detection can be affected by sensitivity of the HBV-DNA assay, the sample size, power of the study and composition of the study populations. In conclusion we agree with Arababadi et al. [1]. Suggestions indicating that OBI is relatively common in high risk cases such as hemodialysis and HIV infected patients. Therefore, the risk of HBV transmission is probable in these groups and screening of high risk groups is recommended.
